# CYP3A4 inhibitors do not influence [^68^Ga]Ga-DOTA-TATE uptake in liver tissue

**DOI:** 10.1186/s13550-023-01034-w

**Published:** 2023-09-22

**Authors:** Youssef Chahid, Faouzi Chahid, Ewoudt van de Garde, Jan Booij, Hein J. Verberne, N. Harry Hendrikse

**Affiliations:** 1 Department of Radiology and Nuclear Medicine, University of Amsterdam, Amsterdam UMC, Location AMC, Meibergdreef 9, 1105 AZ Amsterdam, The Netherlands; 2 Department of Pharmacy, University of Amsterdam, Amsterdam UMC, Amsterdam, The Netherlands; 3 Department of Pharmaceutical Sciences, Utrecht University, Utrecht, The Netherlands; 4 Department of Radiology and Nuclear Medicine, University of Amsterdam, Amsterdam UMC, Amsterdam, The Netherlands

## Background

The use of the positron emission tomography (PET) tracer [^68^ Ga]Ga-DOTA-TATE for somatostatin receptor (SSTR) imaging is common in patients with neuroendocrine tumours (NETs) [1]. PET-SSTR is indicated for various purposes, including initial staging at diagnosis, primary tumour localization, staging before surgery, patient selection for peptide receptor radionuclide therapy (PRRT), and post-PRRT studies to serve as a new baseline 9–12 months after the completion of treatment for future comparisons [1]. Somatostatin analogues (SSAs) are frequently prescribed to NET patients due to their ability to alleviate symptoms associated with NET and to slow down tumour growth [2]. These SSAs play a crucial role in the comprehensive management of NET and are an important component of the treatment strategy for many NET patients [2]. The maximum standardized uptake value (SUV_max_) tumour-to-liver ratio (TLR) of [^68^Ga]Ga-DOTA-TATE can be used as a predictive marker for patient selection in PRRT. This is due to the strong correlation between the efficacy of PRRT and an SUVmax TLR > 2.2 [3]. Furthermore, an SUV_max_ TLR ≥ 8.1 has been associated with extended progression-free survival (PFS) in individuals with NET who are undergoing treatment with SSAs [4]. However, several studies have shown a significant decrease in liver uptake of [^68^Ga]Ga-DOTA-TATE in patients receiving SSAs compared to those not receiving SSAs [5]. Consequently, the use of SSAs can impact the SUVmax TLR, potentially affecting the application of this parameter in clinical practice.

The reduction in liver uptake of [^68^Ga]Ga-DOTA-TATE varied between 10 and 60%, but the precise mechanism underlying this phenomenon remains unclear [5]. It has been suggested that this decrease may be attributed to distinct internalization patterns of SSTRs in normal tissues and tumour cells, resulting in downregulation of SSTRs in the normal liver tissue [6, 7]. On the other hand, a study by Reynaert et al. did not identify any SSTR expression in normal hepatocytes and hepatic stellate cells [8].

Another interesting hypothesis proposes that liver uptake of [^68^Ga]Ga-DOTA-TATE may be influenced by its metabolism in the liver [9]. Limited data indicate that somatostatin analogues exhibit moderate inhibitory effects on cytochrome P450 3A4 (CYP3A4) [10, 11]. This inhibition could potentially reduce the metabolism of [^68^Ga]Ga-DOTA-TATE, thereby affecting its decreased hepatic uptake. Based on these findings, our objective is to investigate the impact of CYP3A4 inhibitors on [^68^Ga]Ga-DOTA-TATE liver uptake.

## Materials and methods

### Study population

This retrospective study, conducted at the Amsterdam University Medical Centers, location AMC, in the Netherlands, received approval from the local Medical Ethics Assessment Committee. The study included a total of 70 patients who underwent a [^68^Ga]Ga-DOTA-TATE PET/CT scan between July 2016 and May 2023. As the administration of SSAs is known to be associated with a decreased [^68^Ga]Ga-DOTA-TATE hepatic uptake [5], patients using SSAs at the time of PET/CT imaging were excluded from the study. The patients were divided into two groups based on their use of CYP3A4 inhibitors. The first group consisted of patients who were taking medications such as clarithromycin, ketoconazole, amiodarone, and verapamil, known inhibitors of CYP3A4, at the time of [^68^Ga]Ga-DOTA-TATE PET/CT imaging [12]. The second group consisted of patients who did not use medication that influences CYP3A4.

### Clinical characteristics

For the statistical analyses, we collected the following data from the electronic health records: sex, age, body mass index (BMI), primary tumour location, presence of a primary tumour, grade and stage of tumour, ki-67 values, injected activity dose and amount of peptide of [^68^Ga]Ga-DOTA-TATE, treatment with [^177^Lu]Lu-DOTA-TATE, and medication use [13].

The procedures for labelling [^68^Ga]Ga-DOTA-TATE, conducting the PET/CT procedure, and performing image analysis have been extensively described elsewhere [13]. In summary, the maximum standardized uptake value (SUV_max_) of [^68^Ga]Ga-DOTA-TATE was calculated on standard iterative reconstructions with a spherical volume of interest (VOI) tool in the primary tumour, the liver (i.e. physiological uptake) and the left psoas major muscle (i.e. background). The following parameters were determined:

- SUV_max_ tumour-to-background ratio (SUV_max_ TBR) = SUV_max_ primary tumour/SUV_max_ psoas major muscle
- SUV_max_ liver-to-background ratio (SUV_max_ LBR) = SUV_max_ liver/SUV_max_ psoas major muscle
- SUV_max_ tumour-to-liver ratio (SUV_max_ TLR) = (SUV_max_ primary tumour)/(SUV_max_ liver)

### Statistical analysis

Patient, tumour, and medication characteristics were assessed using descriptive statistics. IBM SPSS Statistics (version 28, IBM, USA) was utilized for all statistical analyses. Categorical variables were presented as frequency with percentage, while continuous variables were reported as mean ± standard deviation (SD) or median with interquartile range (IQR). Fisher's exact test was employed for categorical variables, whereas unpaired *T*-test or Mann–Whitney U test was used for quantitative variables. All statistical tests were two-tailed, and a *p* value below 5% was considered statistically significant.

## Results

### Study population

A cohort of 70 patients underwent PET/CT scans that met the inclusion criteria and were consequently enrolled in the study. The characteristics of these patients during the PET/CT scan are summarized in Table 1. The majority of the patients (74%, *n* = 52) were male, and 20 patients were prescribed medication that inhibits CYP3A4 enzymes prior to the [^68^Ga]Ga-DOTA-TATE PET/CT. To the best of our knowledge, patients included in the study were taking the following medication, including the averaged prescribed dosages, at the time PET/CT imaging: amiodarone (200 mg/day), verapamil (180 mg/day), clarithromycin (750 mg/day), and ketoconazole (1200 mg/day). Among the CYP3A4 users group, a total of 10 patients exhibited no tumour. No significant differences were observed between the two groups in terms of primary tumour location, primary tumour resection, metastasis, WHO NETs grade, tumour stage, administered [^68^Ga]Ga-DOTA-TATE activity (MBq/kg), and [^68^Ga]Ga-DOTA-TATE peptide amount (ng/kg). These findings, as shown in Table 1, indicate that there were no significant differences in patient or tumour characteristics between both groups.

**Table 1:** Patient characteristics of the study population

Characteristic	No CYP3A4 (%)	CYP3A4 (%)	*p* value
Sex			1.000**
Male	37 (74.0)	15 (75.0)	
Female	13 (26.0)	5 (25.0)	
Age (years)^†^	63.8 ± 9.4	68.0 ± 8.8	0.085^#^
BMI (kg/m^2^)^†^	27.2 ± 3.9	28.9 ± 5.4	0.202^#^
Ki-67 value (%)*	2.0 (1.0–6.7)	3.0 (1.0–17.0)	0.388^¶^
Primary tumour location			0.760**
Pancreas	28 (59.6)	7 (35.0)	
Small bowel	16 (34.0)	3 (15.0)	
Rectum	1 (2.1)	0 (0)	
Stomach	1 (2.1)	1 (5.0)	
Unknown location	1 (2.1)	0 (0)	
No tumour	3 (6.4)	9 (45.0)	
Primary tumour resection			1.000**
Resected	4 (8.5)	1 (9.1)	
Not resected	43 (91.5)	10 (90.9)	
WHO NETs grade			0.134**
I	24 (52.1)	3 (27.3)	
II	23 (47.9)	5 (45.5)	
III	0 (0)	1 (9.1)	
Unknown	0 (0)	2 (18.2)	
Tumour stage			0.353**
I	5 (10.6)	0 (0)	
II	14 (29.8)	3 (27.3)	
III	10 (21.3)	3 (27.3)	
IV	2 (4.3)	2 (18.2)	
Unknown	16 (34.0)	3 (27.3)	
Metastasis			0.181**
Yes	32 (68.1)	5 (45.5)	
No	15 (31.9)	6 (54.5)	
PET/CT			0.359**
Initial	36 (72.0)	3 (15.0)	
Follow-up	14 (28.0)	17 (85.0)	
PET/CT scanner			1.000**
Philips	41 (82.0)	17 (85.0)	
Siemens	9 (18.0)	3 (15.0)	
Activity (MBq/kg)^†^	1.4 ± 0.2	1.4 ± 0.3	0.291^#^
Peptide (ng/kg)^†^	158 ± 66	156 ± 91	0.954^#^
[^177^Lu]Lu-DOTA-TATE therapy			1.000**
Yes	0 (0)	0 (0)	
No	47 (100)	11 (100)	
CYP3A4 inhibitors			NA
Verapamil	NA	10	
Amiodarone	NA	7	
Clarithromycin	NA	2	
Ketoconazole	NA	1	

*Median (interquartile range)

**Fisher’s exact test

^†^Mean ± standard deviation

^¶^Mann–Whitney U test

^#^Unpaired t-test

*NA* Not applicable

### [^68^Ga]Ga-DOTA-TATE uptake

There were no significant differences in the median SUV_max_ TBR between patients who used CYP3A4 inhibiting medication during the [^68^Ga]Ga-DOTA-TATE PET/CT and patients without CYP3A4 inhibitors (Table 2). Similarly, there was no significant difference in the mean SUV_max_ LBR between patients who did not use CYP3A4 inhibiting medication and those who used CYP3A4 inhibitors. Furthermore, the median SUV_max_ TLR also showed no significant difference between the two groups under study.

**Table 2:** SUV_max_ values of patients with CYP3A4 inhibitors during the [^68^ Ga]Ga-DOTA-TATE PET/CT compared to patients without CYP3A4 inhibitor use

Characteristic	No CYP3A4*	CYP3A4*	*p* value^†^
SUV_max_ TBR^‡^	26.6, 18.6–34.1 *n* = 43	28.1, 16.3–60.0 *n* = 10	0.838
SUV_max_ LBR	8.9, 7.2–11.9 *n* = 50	9.2, 7.0–14.1 *n* = 20	0.550
SUV_max_ TLR^‡^	3.0, 1.8–4.7 *n* = 43	3.4, 1.7–5.7 *n* = 10	0.733

*Mean ± standard deviation (median, interquartile range)

^†^Mann–Whitney U test

^‡^Some patients did not have a diagnosed tumour or had already undergone surgical resection of the primary tumour

*TBR* tumour-to-background ratio, *LBR* liver-to-background ratio, *TLR* tumour-to-liver ratio

## Discussion

To the best of our knowledge, this study is the first study investigating the effect of CYP3A4 inhibiting medication on [^68^Ga]Ga-DOTA-TATE uptake. Our findings revealed no significant difference in SUV_max_ LBR between patients on CYP3A4 inhibitors and patients without CYP3A4 inhibiting medication at the time of [^68^Ga]Ga-DOTA-TATE PET/CT imaging. Additionally, there were no significant differences observed in SUV_max_ TBR and SUV_max_ TLR between these two groups.

While the metabolism of [^68^Ga]Ga-DOTA-TATE remains largely unknown [14], substantial evidence from the Erasmus and NETTER-1 studies indicates that [^177^Lu]Lu-DOTA-TATE does not undergo hepatic metabolism. Instead, it is primarily excreted as an intact compound through the renal route [15]. Moreover, [^68^Ga]Ga-DOTA-TOC also follows a similar pattern of being excreted unchanged via the kidneys [16].

Our initial hypothesis suggested that the use of CYP3A4 inhibitors would result in decreased metabolism of [^68^Ga]Ga-DOTA-TATE in the liver, leading to lower hepatic absorption of the tracer. However, our findings indicate that the observed percentage decrease in liver uptake of [^68^Ga]Ga-DOTA-TATE in the literature cannot be explained by the mechanism of CYP3A4 inhibiting. In the literature, the mean percentage decrease in liver uptake of [^68^Ga]Ga-DOTA-TATE after use of SSAs is reported to range from 10 to 60% [5]. If this decrease were solely due to CYP3A4 inhibition, we would have expected to observe a significant difference between the groups in our study. However, no significant differences were found.

The retrospective nature of this study introduces certain limitations, such as the absence of measurements of hepatic uptake of [^68^Ga]Ga-DOTA-TATE before and after the administration of CYP3A4 inhibitors within the same patient. Furthermore, the utilization of various PET/CT scanners throughout the study duration adds complexity to the comparison of SUV_max_ values. Nevertheless, the variation in PET/CT scanners is representative of real-world practice and is commonly acknowledged as a limitation in multicentre clinical trials. To address this issue, we used both tumour-to-background and tumour-to-liver ratios, which minimize discrepancies between different imaging systems and potentially facilitate the generalization of our findings to diverse clinical settings [17].

In conclusion, our study demonstrates that there is no significant effect of CYP3A4 inhibitors on [^68^Ga]Ga-DOTA-TATE liver uptake. Therefore, we can conclude that CYP3A4 inhibition is an unlikely explanation for the observed decrease in hepatic uptake of [^68^Ga]Ga-DOTA-TATE in patients with SSAs.

## Data Availability

The datasets used and/or analysed during the current study are available from the corresponding author on reasonable request.

## Abbreviations

PET/CT: Positron emission tomography computed tomography
SSTR: Somatostatin receptor
NETs: Neuroendocrine tumours
SSAs: Somatostatin analogues
CYP3A4: Cytochrome P450 3A4
VOI: Volume of interest
SUVmax: Maximum standardized uptake value
TBR: Tumour-to-background ratio
LBR: Liver-to-background ratio
TLR: Tumour-to-liver ratio
